# Polyphyllin I exerts anti-hepatocellular carcinoma activity by targeting ZBTB16 to activate the PPARγ/RXRα signaling pathway

**DOI:** 10.1186/s13020-024-00984-0

**Published:** 2024-08-24

**Authors:** Lu shan, Yijun Chen, Guo An, Xiaoyu Tao, Chuanqi Qiao, Meilin Chen, Jiaqi Li, Ruichao Lin, Jiarui Wu, Chongjun Zhao

**Affiliations:** 1 Department of Clinical Chinese Pharmacy, School of Chinese Materia Medica, Beijing University of Chinese Medicine, Beijing, 102488 China; 2 Beijing Key Laboratory for Quality Evaluation of Chinese Materia Medica, School of Chinese Materia Medica, Beijing University of Chinese Medicine, Beijing, 102488 China; 3 Institute of Prescriptions and Syndromes, Guangzhou University of Traditional Chinese Medicine, Guangzhou, China; 4 Key Laboratory of Carcinogenesis and Translational Research (Ministry of Education/Beijing), Department of Laboratory Animal, Peking University Cancer Hospital & Institute, Beijing, 100142 China; 5 Department of Pharmacy, Jinjiang Municipal Hospital, Quanzhou, 362200 Fujian China

**Keywords:** Polyphyllin I, Hepatocellular carcinoma, ZBTB16, PPARγ/RXRα signaling pathway

## Abstract

**Background:**

Studies have reported that polyphyllin I (PPI) had effective anti-tumor activity against hepatocellular carcinoma (HCC). However, the precise molecular mechanism of this action and the direct target remain unclear. The aim of this study was to discover the molecular targets and the exact mechanism of PPI in the treatment of HCC.

**Methods:**

Various HCC cells and Zebrafish xenotransplantation models were used to examine the efficacy of PPI against HCC. A proteome microarray, surface plasmon resonance (SPR) analysis, small molecule transfection, and molecular docking were conducted to confirm the direct binding targets of PPI. Transcriptome and Western blotting were then used to determine the exact responding mechanism. Finally, the anticancer effect and its precise mechanism, as well as the safety of PPI, were verified using a mouse tumor xenograft study.

**Results:**

The results demonstrated that PPI had significant anticancer activity against HCC in both in vitro studies of two cells and the zebrafish model. Notably, PPI selectively enhanced the action of the Zinc finger and BTB domain-containing 16 (ZBTB16) protein by directly binding to it. Furthermore, specific knockdown of ZBTB16 markedly attenuated PPI-dependent inhibition of HCC cell proliferation and migration caused by overexpression of the gene. The transcriptome and Western blotting also confirmed that the interaction between ZBTB16 and PPI also activated the PPARγ/RXRα pathway. Finally, the mouse experiments confirmed the efficacy and safety of PPI to treat HCC.

**Conclusions:**

Our results indicate that ZBTB16 is a promising drug target for HCC and that PPI as a potent ZBTB16 agonist has potential as a therapeutic agent against HCC by regulating the ZBTB16/PPARγ/RXRα signaling axis.

**Supplementary Information:**

The online version contains supplementary material available at 10.1186/s13020-024-00984-0.

## Background

Hepatocellular carcinoma (HCC) is one of the most common malignancies and ranks as the third leading cause of cancer-related death worldwide [1]. China is one of the areas with the highest risk for HCC, contributing 45.3% of all HCC cases worldwide in 2020 [2, 3]. Research has shown that the survival time of over 80% of HCC patients is less than 5 years [4], with surgery currently the first choice and most effective treatment [5]. However, the high recurrence rate for HCC of over 70% over 5 years is the major barrier to prolonging survival and improving the quality-of-life of patients [6]. Traditional Chinese medicine (TCM) has significant advantages in treating HCC [7, 8], with the screening of lead TCM compounds with significant therapeutic effects having great importance for the future development of targeted therapeutic drugs [9].

Zinc finger and BTB domain-containing 16 (ZBTB16, a.k.a. PLZF) is a protein-coding gene that was first identified in a patient with acute promyelocytic leukemia (APL) [10]. The gene codes for zinc finger transcription factors and affects a diverse number of signaling pathways related to the cell cycle, differentiation, and programmed cell death pathways [11–14]. Previous reports have demonstrated that ZBTB16 is under-expressed or silenced in multiple tumor tissues or various cancer types, including HCC [15, 16]. Furthermore, published results have indicated that ZBTB16 in HCC was closely related to the level of alkaline phosphatase in patients while showing good diagnostic value in distinguishing tumors from normal tissues, suggesting its potential as an HCC biomarker [15].

Polyphyllin I (PPI, PubChem CID: 129316759) is an anticancer molecular extracted from Rhizoma Paridis that has significant anticancer effects in various cancers including HCC [17–19]. Despite this, its direct targets and the potential molecular mechanism remain to be elusive. Therefore, it is important to perform comprehensive in-depth research on the mechanisms of PPI, facilitating the development of novel treatments to improve patient outcomes.

**Fig. 1:**
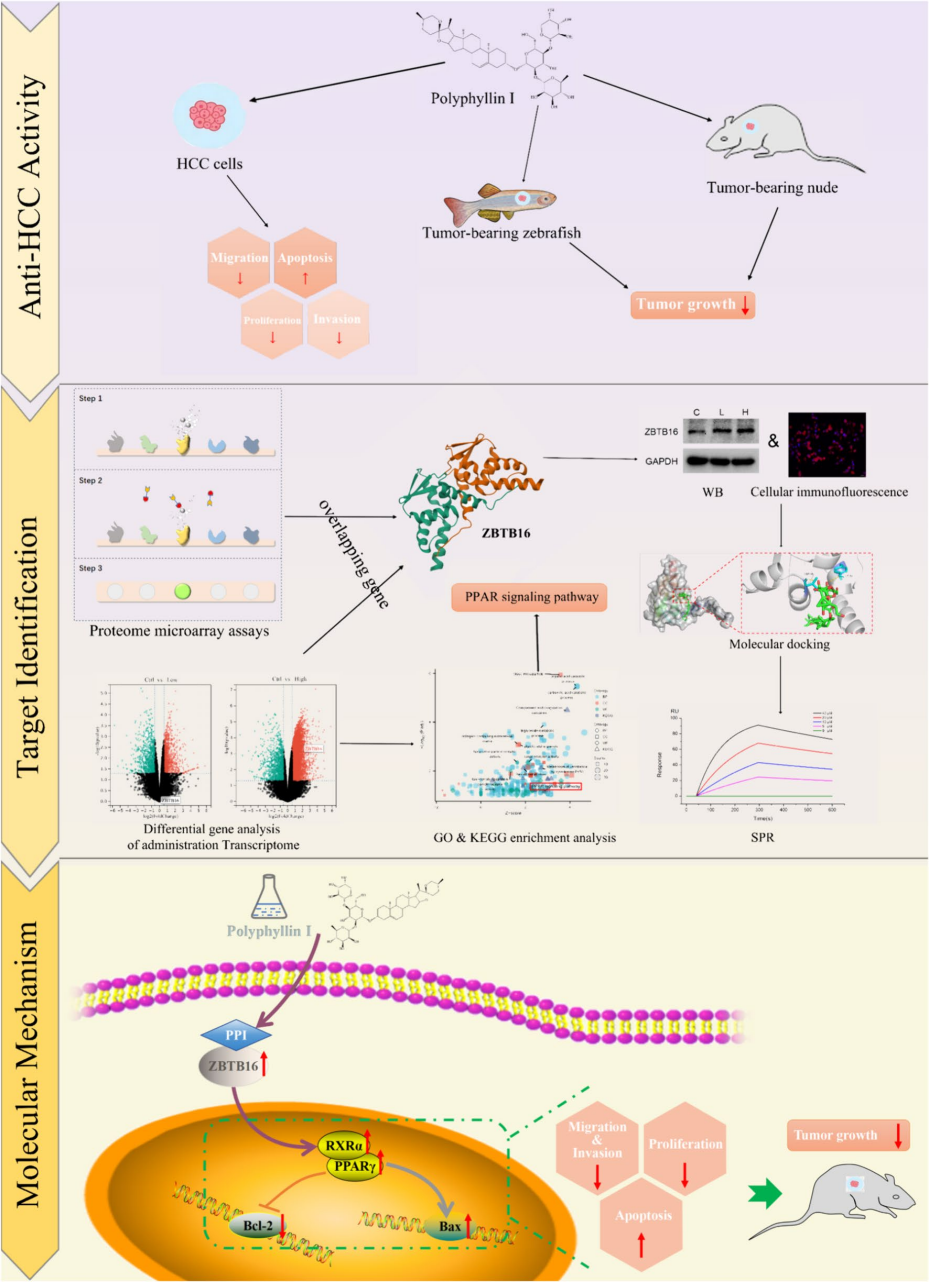
Workflow of the current study

The current study investigated the key direct target of PPI using proteomic microarrays that was confirmed using surface plasmon resonance (SPR) analysis. Further investigation was conducted using cell transcriptomics to identify the signaling pathway of PPI that inhibited key direct targets associated with HCC, which was then validated in both in vitro and in vivo experiments. We also evaluated the safety of therapeutic doses of PPI that provided a solid foundation for the development of PPI as a novel targeted cancer treatment drug. The workflow of this study is shown in Fig. 1.

## Methods

### Cell proliferation assay

The primary HCC cells (Hep3B 2.1–7 and SK-Hep-1) were purchased from Procell (Wuhan, China) and cultured in medium (Procell) supplemented with 10% FBS (Corning, VA, USA) and 1% penicillin–streptomycin (Gibco, Waltham, Massachusetts, USA). The cells were seeded in 96-well culture plates at a density of 1 × 10^4^ cells/well. The next day, 200 mL of fresh medium with different concentrations of PPI (Chengdu Herbpurify Co., Ltd, Chengdu, China) were added to the cells, followed by incubation for 24 h. At the end of the experiment, the cell counting kit-8 (CCK-8; Beyotime, Beijing, China) was used to assess the effect of PPI on cell proliferation, with the absorbance at 450 nm measured in a microplate reader (Synergy H1, Biotek, Vermont, USA) to calculate the dose–effect curve prepared using GraphPad Prism 8.0 (GraphPad Software, La Jolla, USA).

### Apoptosis analysis

Based on the results of the CCK8 experiment, the drug concentration for the subsequent in vitro cell experiments was determined to be 2.5 μg/mL (low-dose group, L) and 5 μg/mL (high-dose group, H). The cells were cultured and then administered PPI as described above. At the endpoint of the experiment, 1 × 10^7^ cells from the different groups were collected, incubated with 5 μL of Annexin V-fluorescein isothiocyanate (FITC) at room temperature in the dark for 15 min and then incubated with 5 μL of propidium iodide (PI). Finally, the stains were analyzed using a flow cytometer and the Pharmingen^™^ FITC Annexin V Apoptosis Detection Kit I according to the manufacturer’s instructions (BD Biosciences, Franklin Lakes, USA).

### Transcriptome analysis by RNA-seq

Hep3B 2.1–7 cells (1 × 10^8^) from the different treatment groups and control were collected. The transcriptome analysis by RNA-seq was conducted according to our previous study [20, 21]. TRIzol reagent (R0016, Beyotime, Beijing, China) was used to extract the total RNA, which was then used to prepare the RNA-seq transcriptome library using the TruSeqTM RNA sample preparation Kit (Illumina, CA, USA). The Illumina NovaSeq 6000 platform (Illumina, San Diego, California) was used to process the short-sequenced fragments.

### Western blotting

The total protein of the cells in the different groups was extracted using a protein extraction kit and the protein concentration estimated by the bicinchoninic acid (BCA) method (Biorigin, Beijing, China). SDS-PAGE (Millipore, Darmstadt, Germany) was used to separate the proteins, which were then transferred electrophoretically to a polyvinylidene difluoride membrane, blocked in 5% milk/Tris-buffered saline/Tween buffer and incubated overnight at 4 ℃ with the primary antibody. The following primary antibodies were used against GAPDH (Proteintech, Wuhan, China; Cat No. 60004-1-Ig, Lot #10028230, 1:1000), Caspase 3 (Proteintech, Cat No. 19677-1-AP, Lot #10018674, 1:1000), cleaved Caspase 3 (Affinity, Jiangsu, China; Cat No. AF7022, Lot #15z0096, 1:1000), ZBTB16 (Santa Cruz, CA, USA; Cat No. sc-28319, Lot #I1022, 1:500), PPARγ (Affinity, Cat No. AF6284, Lot #58o2063, 1:1000), RXRα (Affinity, Cat No. DF8459, Lot #00094775, 1:1000), Bcl2 (Affinity, Cat No. AF6139, Lot #11o9905, 1:1000), and Bax (Affinity, Cat No. AF0120, Lot #44q6915, 1:1000). The membranes were then incubated at room temperature for 90 min with the secondary antibodies (HRP conjugated anti-rabbit or anti-mouse IgG). Finally, the result of Western blotting was visualized using the ChemiDocTM MP Imaging System (Bio-Rad, State, USA; 734BR2920, USA) and analyzed using Image J software (NIH, Bethesda, MD, USA).

### Xenograft tumor assay on zebrafish

The zebrafish embryos were obtained from spawning adults (5–8 months old) and raised at 28.5 °C in zebrafish embryo culture medium until 48 h after fertilization (hpf, hours after fertilization). The healthy zebrafish larvae were selected and microinjected with Hep3B 2.1–7 cells stained with CM-Dil (Beyotime, city, China) and then divided randomly into model, experimental, and control groups. After 24 h, the culture medium of the experimental groups was replaced with a culture medium containing different concentrations of PPI (low-dose group: 0.3 μg/mL, high-dose group: 0.6 μg/mL). An aliquot of 100 ng/ml of sorafenib (MCE, Shanghai, China) was added to the positive group [22, 23]. After 48 h, images were obtained using a Zeiss Axio Zoom V16 stereo fluorescent dissecting microscope (Carl Zeiss, Jena, Germany), and the fluorescence integrated density was calculated by Image J.

### Proteome microarray assays

Arrayit HuProt^™^ 20 K human proteome microarrays (CDI Laboratories, MD, USA) were used and the experiment conducted according to the operating standard for chip detection. The microarrays were incubated with 40 mM biotinylated-PPI or free-biotin for 1 h at room temperature. After washing, 30 mL of Cy5 Streptavidin solution (1:100, Sigma-Aldrich, MO, USA) was added. The data were obtained by Genepix 4000B (Axon Instruments, Sunnyvale, CA, USA) and analyzed by GenePixTM Pro 6.0.

### Surface plasmon resonance (SPR) analysis

The binding affinity of PPI to recombinant human ZBTB16 was determined at 22 °C using OpenSPR^™^ (manufacturer, city/state, country). Briefly, ZBTB16 proteins in PBS were immobilized on the sensor after activation by 200 μL EDC/NHS (1:1) in a water solution. Different concentrations of PPI were prepared in a running buffer (1% DMSO + HEPES). The binding time was 240 s, the dissociation time was 400 s, and the flow rate was 20 mL/min. The data were analyzed using TraceDrawer (Ridgeview Instruments ab, Vänge, Sweden).

### Transfection of plasmids and small-interfering RNA

The siRNA oligonucleotides of ZBTB16 and Flag-tagged ZBTB16 plasmids were obtained from Jingmiga Technology Co. Ltd (Beijing, China). The Hep3B 2.1–7 cells were cultured to about 50% confluence before transfection. The siRNA and plasmids dissolved in an appropriate volume of MEM without the serum and antibiotics were incubated with appropriate transfection reagent and added to the cultured cells for 6 h according to the manufacturer’s instructions. The medium was then changed to MEM containing 10% FBS and antibiotics. At the end of the experiments, the cell samples were collected for further analysis.

### Mouse tumor xenograft studies

Specific pathogen-free (SPF) BALB/c nude mice (4–6 weeks old) were purchased from Beijing SiPeiFu Biotechnology Co., Ltd., and housed and fed in an SPF animal laboratory. The animal experiment was approved by the Animal Ethics Committee of Beijing University of Traditional Chinese Medicine (Animal Protocol No. BUCM-4-2021032003-1091). After a week of adaptive feeding, the mice were injected subcutaneously with cells (1 × 10^7^ in 100 μL of PBS) and then randomized into treatment and control groups (eight mice per group), until the tumors reached a volume of 50 mm^3^. Mice in the experimental groups were treated with 4 mg/kg (L) or 8 mg/kg (H) of PPI [24, 25] and the positive and model group with sorafenib (30 mg/kg) [17] or PBS, respectively. Each mouse was injected twice a week during the trial. The tumor size and body weight were monitored 2 or 3 times per week. The mice were anesthetized using isofluorane gas, followed by collection of the tumor xenografts, main organs, and blood samples.

### Statistical analysis

All the statistical analyses were performed using GraphPad Prism 8.0 software. The results were expressed as mean ± SD based on a minimum of three independent repeated experiments. Dead animals were eliminated from the data analysis. Comparison between the two groups was conducted using Student’s t-test, while multiple group comparisons were conducted using one-way ANOVA. A *p* value < 0.05 indicated a statistically significant difference.

## Results

### *PPI exerts anticancer activity *in vivo* and *in vitro

**Fig. 2:**
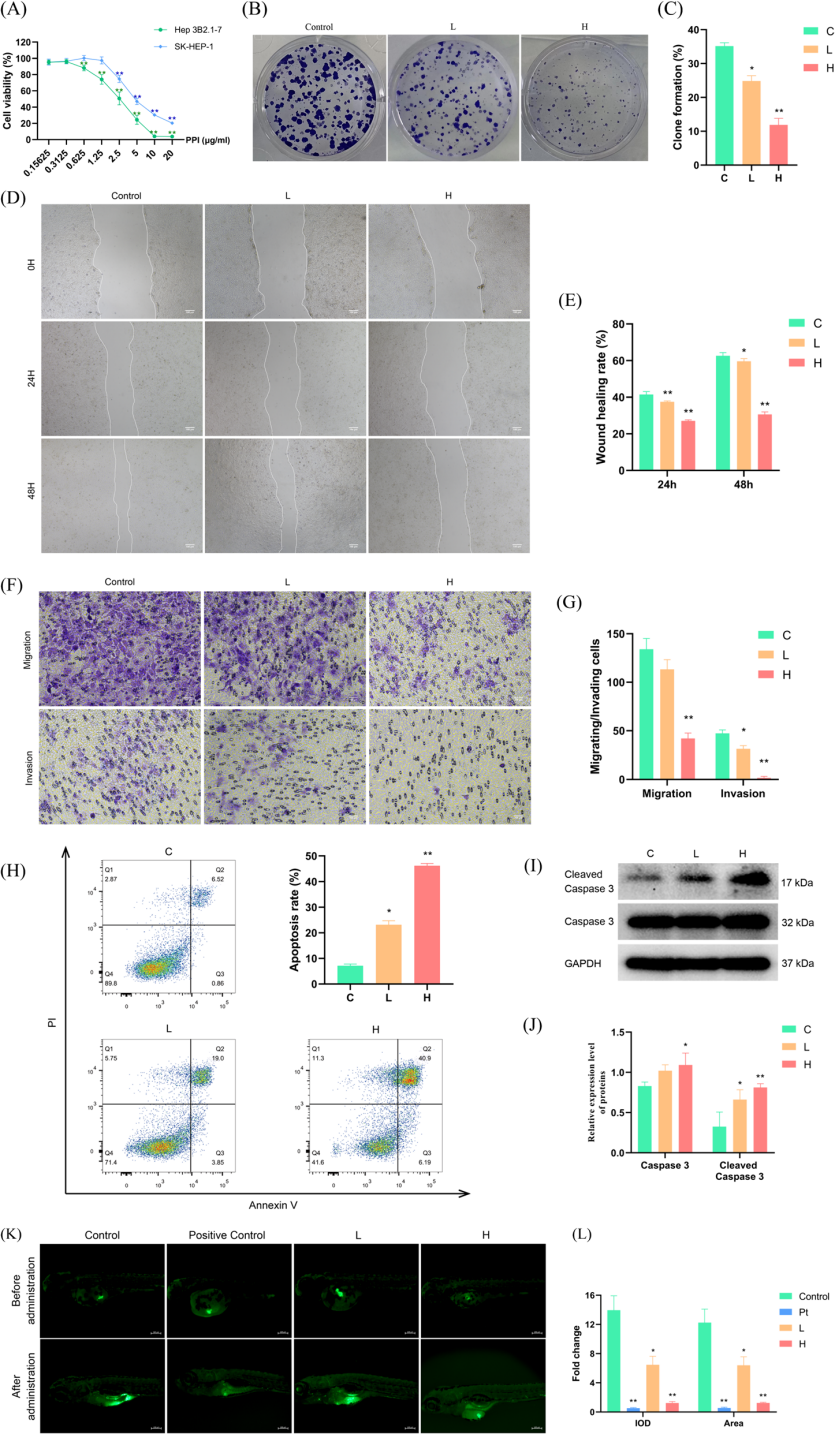
PPI has anticancer activity in vivo and in vitro. **A** CCK8 assay of Hep3B2.1–7 and SK-HEP-1 cells treated with PPI (n = 3). **B**, **C** Colony formation assay of HCC cells treated with PPI (n = 3; L, 2.5 μg/mL PPI; H, 5 μg/mL PPI). **D**, **E** Wound healing assay of HCC cells treated with PPI (n = 3; L, 2.5 μg/mL PPI; H, 5 μg/mL PPI). **F**, **G** Transwell assay of HCC cells treated with PPI (n = 3; L; 2.5 μg/mL PPI; H, 5 μg/mL PPI). **H** Annexin V–FITC dual staining assay of HCC cells treated with PPI (n = 3; L, 2.5 μg/mL PPI; H, 5 μg/mL PPI). **I**, **J** Change in cleaved caspase 3 in HCC cells treated with PPI (n = 3; L, 2.5 μg/mL PPI; H, 5 μg/mL PPI). **K**, **L** PPI significantly inhibited the tumor growth of zebrafish xenograft models injected with Hep3B cells (n = 6; Positive control, 100 ng/mL sorafenib; L, 0.3 μg/mL PPI; H, 0.6 μg/mL PPI). (* *p* < 0.05, ** *p* < 0.01, *** p < 0.001)

To assess the antiproliferative function of PPI on HCC, the Hep3B 2.1–7 and SK-HEP-1 cell lines were treated with different concentrations of PPI. As shown in Fig. 2A–C, PPI markedly inhibited cell viability and colony formation ability in a concentration-dependent manner, especially in Hep3B cells (IC_50_ of Hep3B was 2.412 μg/mL and SK-Hep-1 was 5.059 μg/mL). Furthermore, wound healing and transwell invasion assays were performed to assess the effects of PPI on the migration and invasion of Hep3B cells, respectively. The results showed that PPI caused a significant decline in the migration of Hep3B cells and prevented the invasion of Hep3B cells in a concentration-dependent way (Fig. 2D–G). The flow cytometry results indicated that PPI also induced apoptosis in a concentration-dependent way (Fig. 2H), a finding that was confirmed by an elevated expression of cleaved caspase 3 (Fig. 2I, J). Similarly, PPI treatment suppressed tumor growth in the xenograft tumors of Hep3B cells in the zebrafish (Fig. 2K, L).

### ZBTB16 was identified as a direct target of PPI

**Fig. 3:**
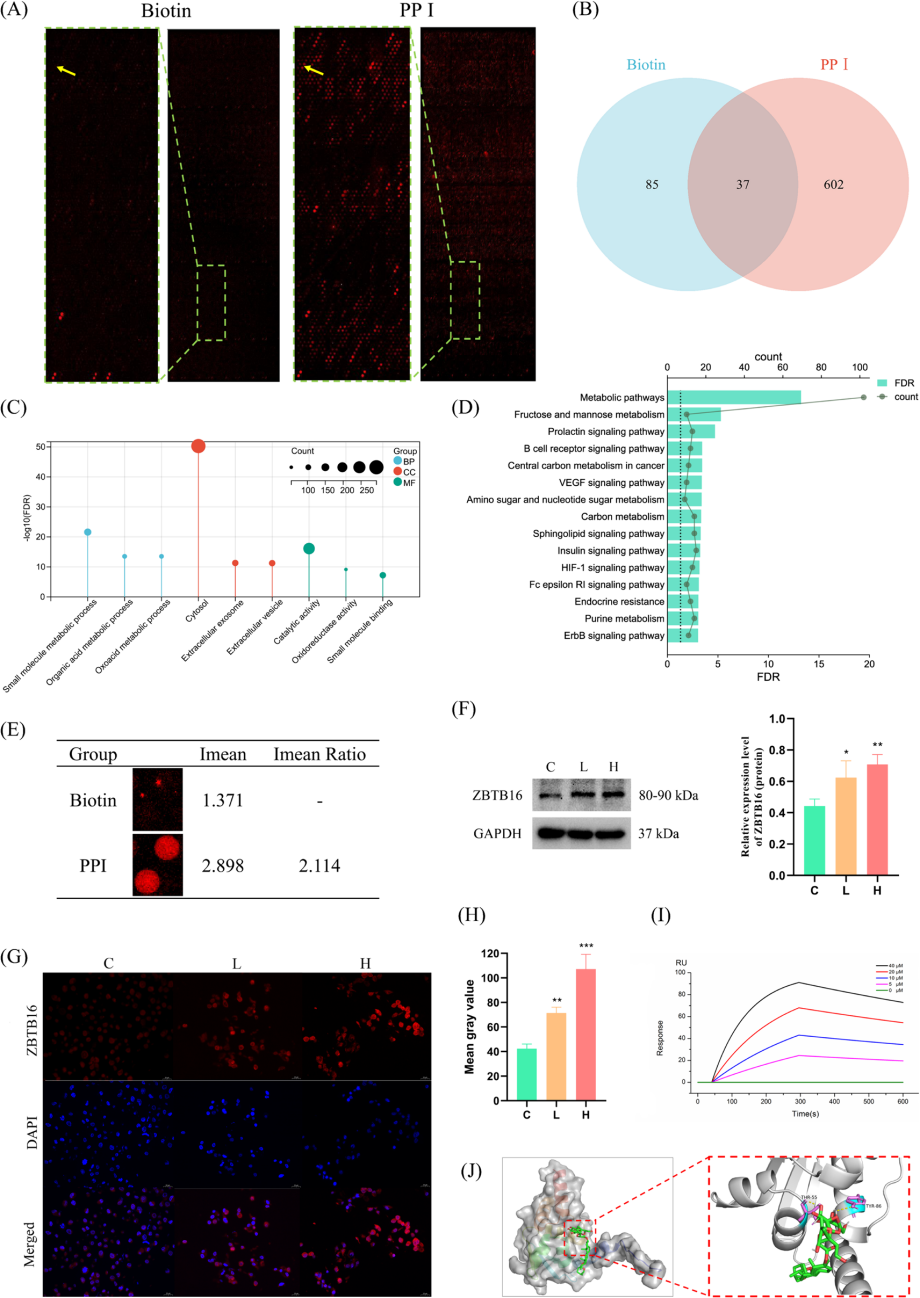
ZBTB16 was identified as a direct target of PPI. **A** HuProt^™^20 K Proteome microarray chip indicating the protein targets binding to PPI. The yellow arrow points to the location of the ZBTB16 protein. **B** Venn diagram of the protein targets binding to biotin and PPI. **C**, **D** GO and KEGG enrichment analysis of the specific binding proteins of PPI. **E** The signal intensity of the ZBTB16 protein in the HuProt^™^20 K proteome microarray chip. **F**–**H** Changes in the expression of the ZBTB16 protein in HCC cells treated with PPI (n = 3; L, 2.5 μg/mL PPI; H, 5 μg/mL PPI). **I** SPR analysis of binding affinity between PPI and the ZBTB16 protein. **J** Molecular docking analysis of PPI binding to the ZBTB16 protein to predict the binding site. (* *p* < 0.05, ** *p* < 0.01, *** *p* < 0.001)

To investigate the potential action target of PPI in HCC, we labeled PPI with biotin and a fluorescent probe, and then performed a HuProt^™^20 K proteome microarray chip to detect the protein binding target to PPI. A total of 602 proteins showed significant specific interaction with PPI on the chip (Fig. 3A, B) that might have contributed to its biological anti-cancer activity. These 602 proteins were significantly enriched in GO entries such as the cytosol (Fig. 3C) and signaling pathways such as metabolic pathways (Fig. 3D).

To accurately identify potential targets of PPI, we conducted a series of analytical steps. Initially, we performed a differential gene analysis on the LIHC cohort from the TCGA database to identify significant gene expression differences between tumor and normal tissues (Figure S1A). Subsequently, we compared the transcriptomic data of HCC cells with and without PPI treatment to screen for genes whose expression significantly changed after the action of PPI (Figure S1B). We then intersected these two sets of differential genes with the potential binding target proteins of PPI predicted by chip technology, thereby identifying 30 potential targets at the center of the intersection (Figure S1C). Furthermore, we conducted Kaplan-Meier survival analysis on these genes and used WB technology to test whether the protein expression levels of genes with potential prognostic value for HCC patients changed with the dosage of PPI (Figure S1D). After this series of comprehensive analyses, we ultimately selected ZBTB16 as a candidate for further research because it not only showed significant prognostic relevance among all potential targets but also had a clear association with the action of PPI.

As show in Fig. 3E, the original signal intensity of the ZBTB16 protein on the PPI-chip was significantly higher than that on the biotin chip, indicating binding of PPI to ZBTB16 in HCC cells. Furthermore, the results of Western blotting and the cellular immunofluorescence assays indicated that PPI may potentially mediate its activities by binding to ZBTB16 proteins (Fig. 3F–H). SPR was then performed with the results demonstrating a high affinity between the recombinant human (rh) ZBTB16 protein and PPI, with a dissociation constant (K_D_) of 5.5 µM (Fig. 3I). This finding was consistent with the results of molecular docking that showed the binding energy of the two was -10.4 cal/mol, indicating that PPI may interact with THR-55 and TYR-86 of ZBTB16 (Fig. 3J).

### Correlation between ZBTB16 and HCC

**Fig. 4:**
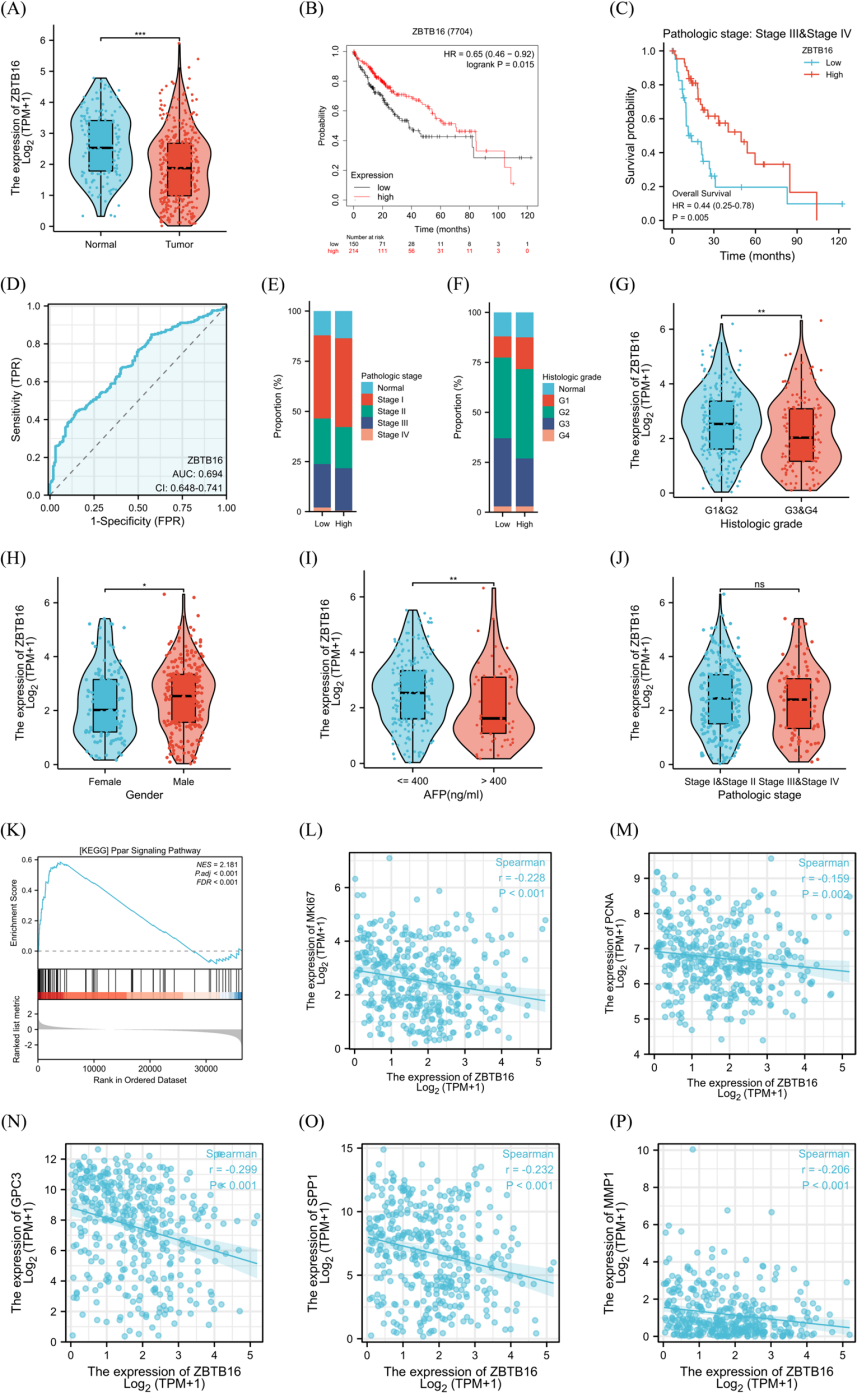
ZBTB16 is inhibited significantly in HCC and is associated with an improved prognosis in patients with the malignancy. **A** ZBTB16 mRNA expression is downregulated in HCC tissues compared with that in the normal tissue groups of the TCGA and GTEx datasets. **B** Kaplan–Meier survival analysis on the association between ZBTB16 expression and overall survival of patients. **C** Kaplan–Meier survival analysis on the association between ZBTB16 expression and overall survival of patients with stage III-IV HCC in the TCGA-LIHC dataset. **D** Diagnostic receiver operating characteristics curves of ZBTB16 in HCC. **E** Composition of the pathologic stage of the ZBTB16-high (n = 173) and -low groups (n = 173) in the TCGA-LIHC dataset. **F** Composition of the histologic grade of the high-ZBTB16 (n = 173) and low-ZBTB16 groups (n = 173) in the TCGA-LIHC dataset. **G**–**J** Comparison of ZBTB16 mRNA expression in the different HCC clinical groups. **K** The GSEA results indicated that the PPAR signaling pathway was enriched significantly in the high-ZBTB16 group. **L**–**P** Correlation analysis of ZBTB16 and mRNA expression levels in the indicated genes in the TCGA-LIHC dataset. (* *p* < 0.05, ** *p* < 0.01, *** *p* < 0.001)

To validate that ZBTB16 was a potential therapeutic target for HCC, we first compared the expression levels of ZBTB16 mRNA in HCC tissue and normal tissue in the TCGA and GTEx datasets. This showed that compared with normal tissue, the mRNA expression level of ZBTB16 in tumor samples was downregulated significantly (Fig. 4A). K-M survival analysis demonstrated that the expression of ZBTB16 was related closely to overall survival (OS) of the HCC patients, with high expression indicating better survival (Fig. 4B), especially in patients with stages III- IV cancers (Fig. 4C). The area under the curve (AUC) of the diagnostic receiver operating characteristic (ROC) of ZBTB16 in HCC was 0.694 (95% CI, 0.648–0.741), indicating a good diagnostic value for HCC (Fig. 4D). Comparison of the composition of HCC patients in the high- and low-ZBTB16 groups showed that the proportion of patients with stage III-IV and grade 3–4 in the low group was higher than that in the high group (Fig. 4E, F). The correlation analysis between ZBTB16 and the clinical variables revealed a strong correlation between its expression and histological grade, gender, and alpha-fetoprotein (AFP) level (Fig. 4G, I), but no correlation with pathological grade (Fig. 4J). To further investigate the functional pathway of ZBTB16 regulation in HCC, we conducted differential gene analysis (GSEA) based on logFC of ZBTB16 high and low group differential genes. It was found that the PPAR signaling pathway was activated significantly in the high group (Fig. 4K). The correlation scatter plot also showed that the expression of ZBTB16 correlated negatively with the expression of cell proliferation-related genes (including Ki67 and PCNA) and HCC diagnostic markers (including GPC3 [26], OPN [27], and MMP1 [28]) (Fig. 4M–Q).

### *ZBTB16 inhibits HCC cell metastasis and growth *in vitro

**Fig. 5:**
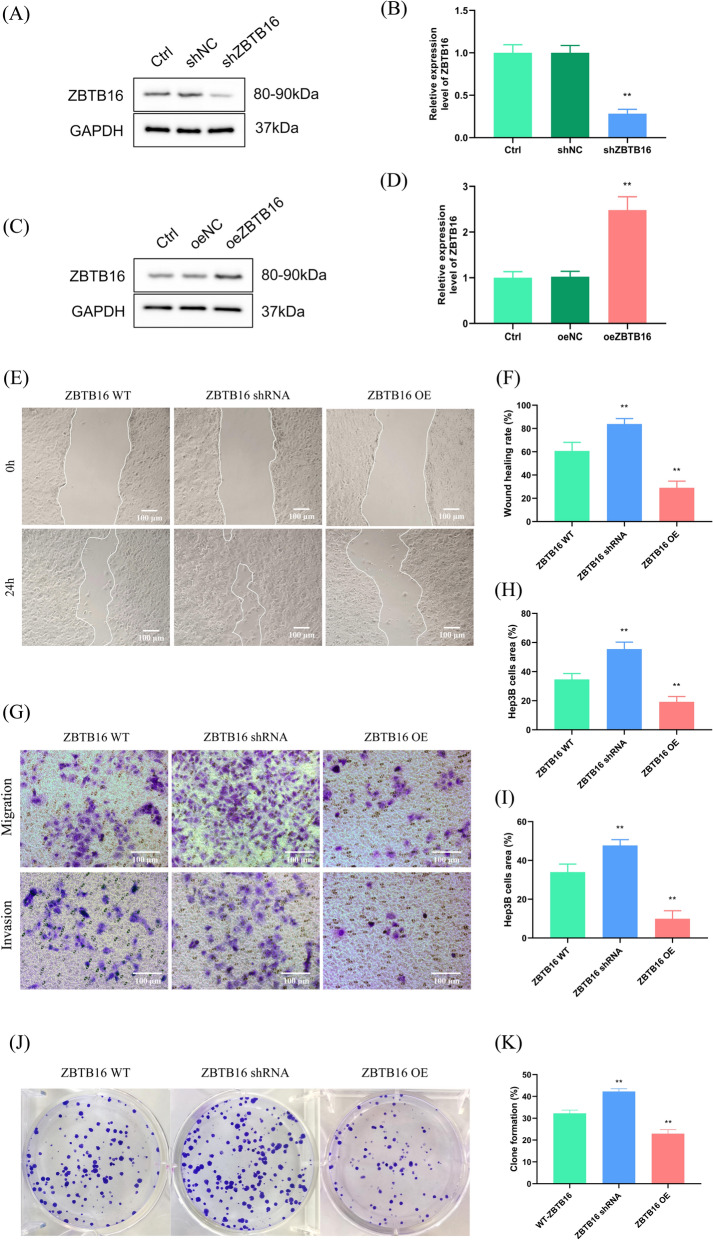
ZBTB16 inhibits cancer cell metastasis and growth in vitro. **A**–**D** ZBTB16 knockdown and overexpressing cell lines were constructed (n = 3). **E**, **F** Wound healing assay using ZBTB16 knockdown and overexpression cell lines (n = 3). **G**–**I** Migration and invasion ability in ZBTB16 knockdown and overexpression cell lines (n = 3). **J**, **K** Cloning formation assay in ZBTB16 knockdown and overexpression cell lines (n = 3). (* *p* < 0.05 and ** *p* < 0.01)

To investigate the function of ZBTB16 in HCC, we constructed knockdown and overexpression cell lines (Fig. 5A–D). The wound healing results showed that ZBTB16 knockdown significantly increased the migration activity of Hep3B2.1–7 cells in vitro. In contrast, ZBTB16 overexpression inhibited the migration of Hep3B2.1–7 cells (Fig. 5E, F). Similar results were observed in the transwell assay based on the characteristics of migration and invasion ability (Fig. 5G, I) of Hep3B2.1–7 cells (Fig. 5J, K). In addition, the inhibitory effect of ZBTB16 on the proliferation of Hep3B2.1–7 cells was confirmed by the results of the plate cloning experiment.

### PPI inhibits HCC by targeting ZBTB16

**Fig. 6:**
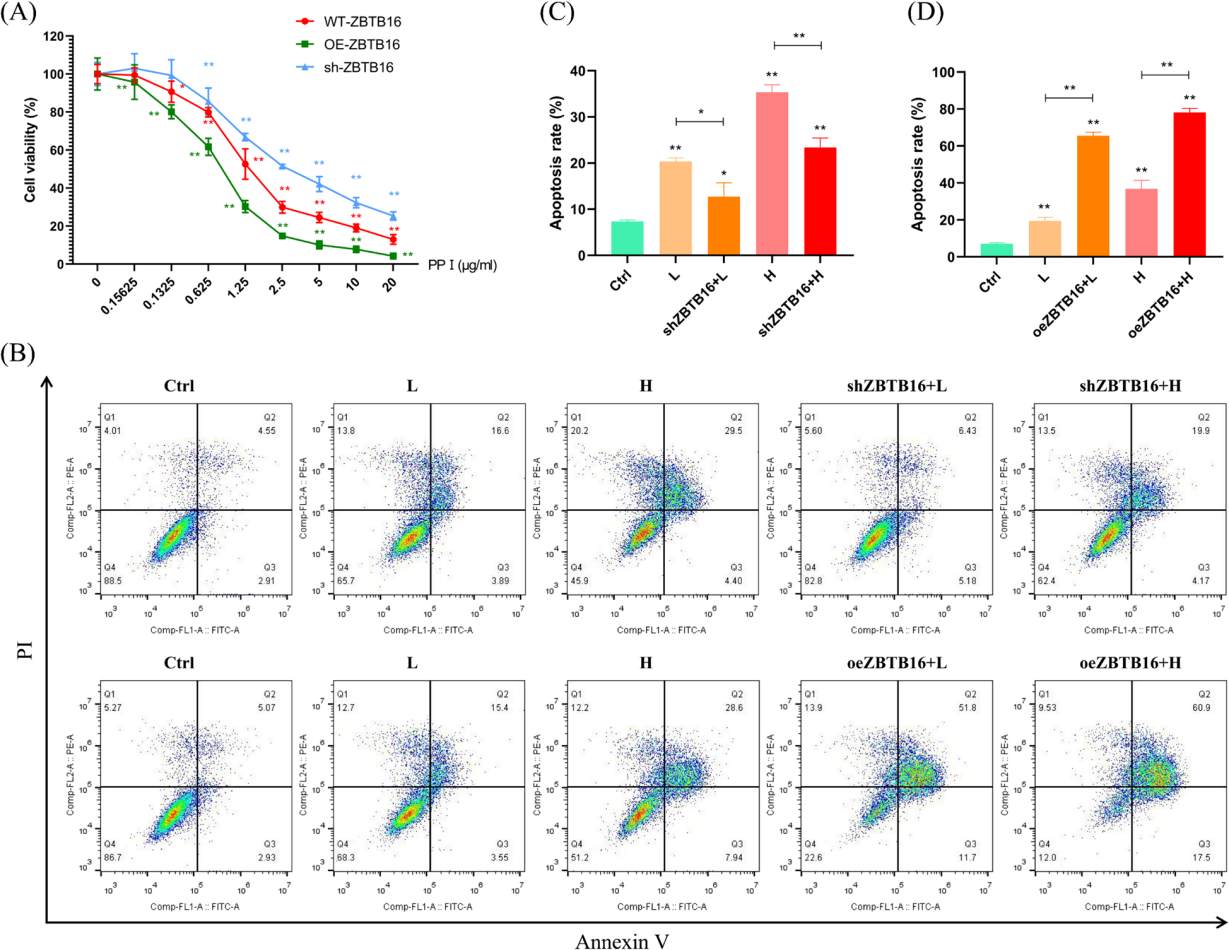
PPI inhibits HCC by targeting ZBTB16. **A** CCK8 assay of ZBTB16 knockdown and overexpression cell lines with PPI (n = 3). **B**–**D** Annexin V–FITC dual staining assay of ZBTB16 knockdown and overexpression cell lines with PPI (n = 3). (L, 2.5 μg/mL PPI; H, 5 μg/mL PPI) (* *p* < 0.01, ** *p* < 0.001)

To further confirm the important role of ZBTB16 in the anticancer activity of PPI, rescue experiments related to ZBTB16 were designed based on the inhibition of PPI in HCC cells. The results of the CCK8 assay showed that at the same dosage, the anti-proliferation effect of PPI on cells with ZBTB16 overexpression was enhanced significantly, while ZBTB16 knockout improved the inhibition of PPI in cells (Fig. 6A). Similar results of cell apoptosis were observed. As shown in Fig. 6B–D, ZBTB16 knockdown significantly abolished the effect of PPI-induced cell apoptosis. In contrast, cell apoptosis of the ZBTB16 overexpressing cell line treated with PPI was significantly higher than that of the PPI group, indicating that ZBTB16 had a key contribution in the anti-cancer effects of PPI.

### Transcriptome analysis revealed the involvement of the PPAR signaling pathway in the anticancer activity of PPI

**Fig. 7:**
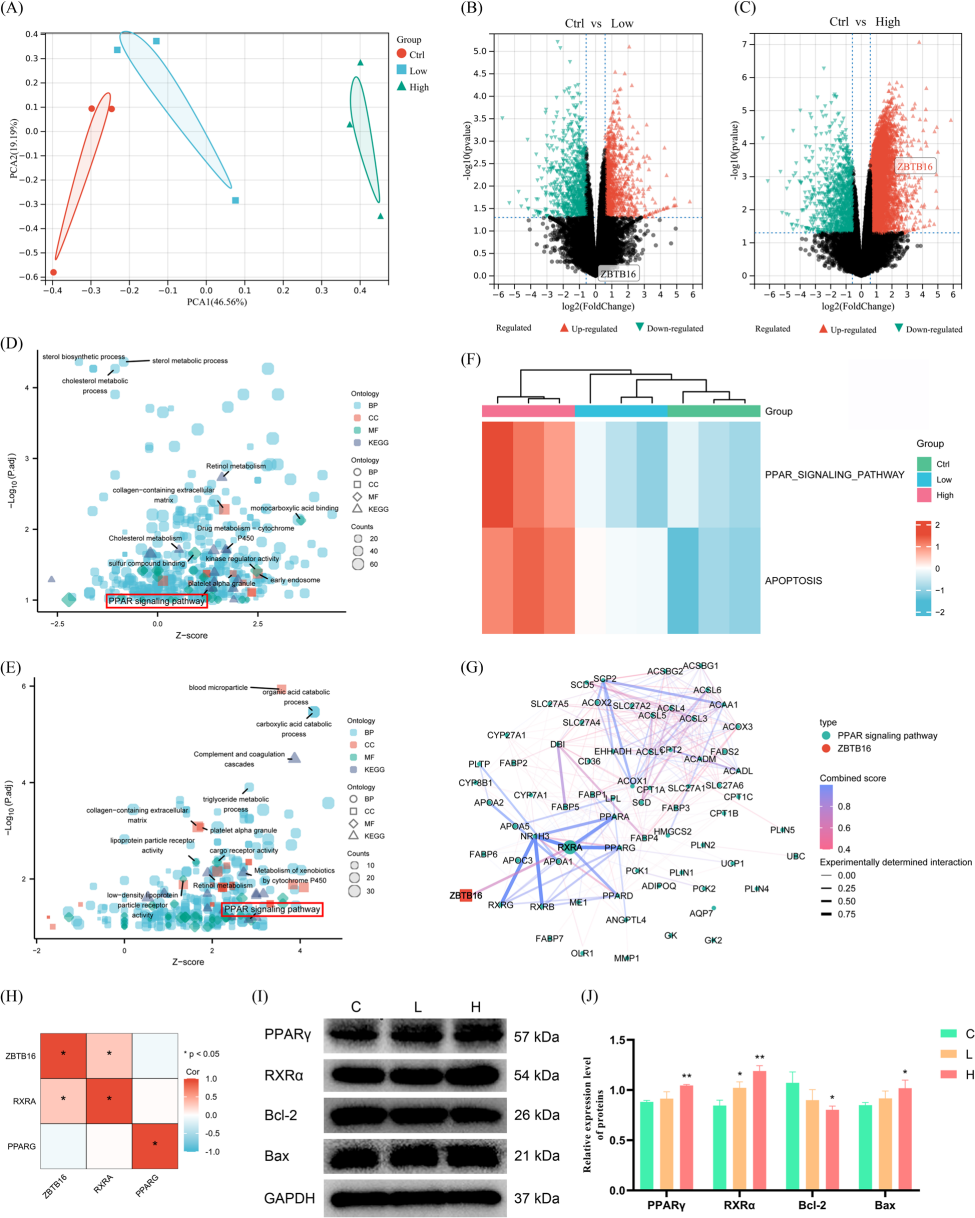
Transcriptome analysis showing the involvement of the PPAR signaling pathway in the anticancer activity of PPI. **A** PCA of transcriptomes with or without different concentrations of PPI represented in a two-dimensional space. **B**, **C** Volcano plot of differential genes in HCC cells induced by low- and high-dose PPI treatment. **D** Bubble plots for the GO and KEGG enrichment analysis of differentially expressed genes induced by low-dose PPI. **E** Bubble plots for the GO and KEGG enrichment analysis of differentially expressed genes induced by high-dose PPI. **F** Heat map showing the GSVA enrichment score for apoptosis and the PPAR signaling pathway in the transcriptome of HCC cells treated with PPI. **G** PPI network of the ZBTB16 and PPAR signaling pathways. **H** Heatmap showing the correlation between the mRNA expression level of ZBTB16, PPARγ, and RXRα. **I**, **J** The regulatory effect of PPI on the PPAR signaling pathway in HCC cells (n = 3; L, 2.5 μg/mL PPI; H, 5 μg/mL PPI). (* p < 0.05, ** *p* < 0.01, *** *p* < 0.001)

To determine the molecular mechanism associated with the anticancer activity of PPI, RNA sequencing of Hep3B cells was conducted. A principal component analysis (PCA) was conducted to detect the clustering trend in the multidimensional data, with the results indicating a distinct segregation between the low and high-PPI and control groups. Compared to the control group, there was a shift in principal component 1 (PC1) and PC2 in PPI (Fig. 7A). Differential gene analysis identified 1032 and 3950 genes as significantly differentially expressed genes (DEGs) in the low and high groups, respectively (| logFC |≥ 1, *p* < 0.05) (Fig. 7B, C). ZBTB16 was upregulated significantly in the high group (Fig. 7C). To obtain an insight into the global patterns of the effects of DEGs on biological processes, we performed GO and KEGG analyses. Briefly, the results showed that DEGs in both the high and low groups were enriched significantly in the PPAR signaling pathway (*p* < 0.05) (Fig. 7D, E). To gain a more comprehensive understanding of the pathways altered in response to PPI, a heatmap of Gene Set Variation Analysis (GSVA) was generated from the transcriptome data. As shown in Fig. 7F, PPI may exert its anticancer activity by activating the PPAR signaling pathway and inducing apoptosis. Through the protein–protein interaction network, it was found that in the PPAR signaling pathway, ZBTB16 only interacts with RXRα, which regulates cell apoptosis by forming heterodimers with PPARγ (Fig. 7G) [29–31]. Based on the TCGA-LIHC dataset, we also showed that ZBTB16 was significantly and positively correlated with RXRα, but not with PPARγ (Fig. 7H). The WB results demonstrated that PPI activated the PPAR signaling pathway by increasing RXRα and PPARγ significantly, and upregulated Bax but decreased Bcl-2 protein (*p* < 0.05), leading to apoptosis in Hep3B cells (Fig. 7I, J).

### *PPI inhibits the growth of HCC *in vivo* without causing significant host toxicity*

**Fig. 8:**
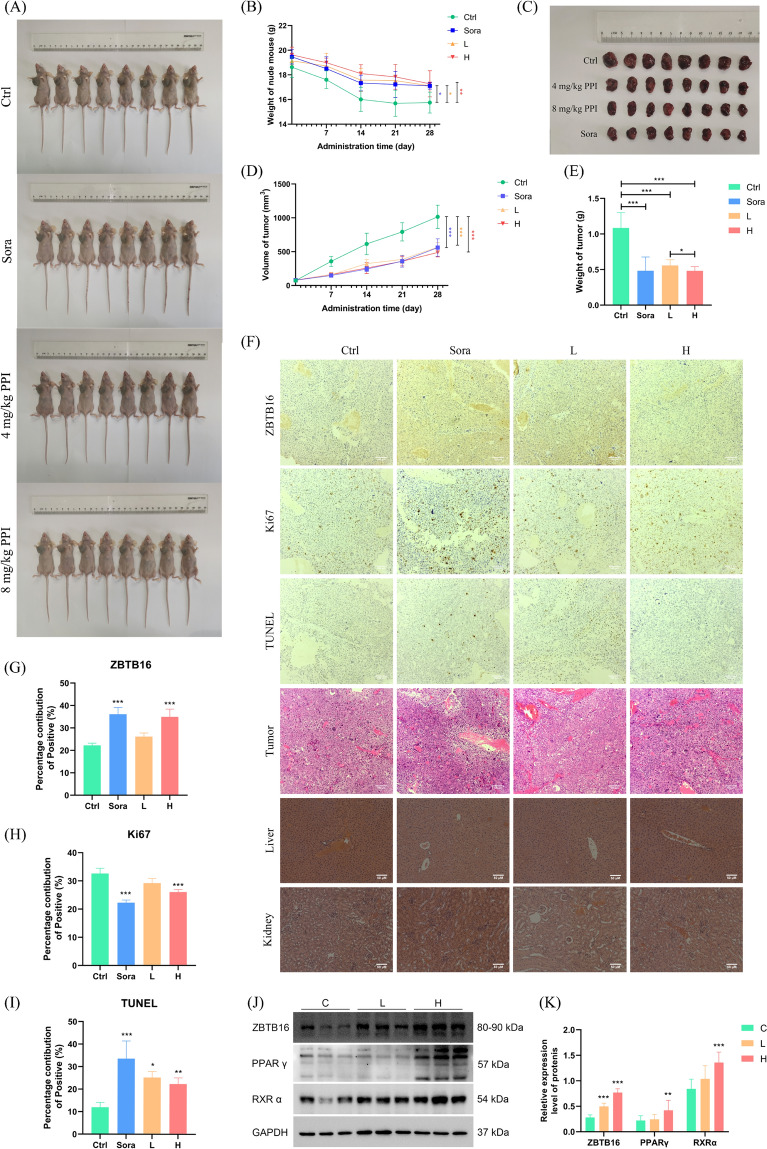
PPI inhibits the growth of HCC in vivo without causing significant host toxicity. **A** Photos of nude mice in each group at the end of the experiment. **B** Graph showing the change in body weight of the nude mice in each group during administration of PPI. **C**–**E** PPI significantly inhibited tumor growth in the mouse xenograft models injected with Hep3B (n = 8). **F**–**I** Immunohistochemical and Tunel assay of tumor bodies and liver and kidney sections in the nude mice in each group. **J**, **K** PPI significantly activated the ZBTB16-mediated PPAR signaling pathway (n = 3). (C, control; Sora, 30 mg/kg sorafenib; L, 4 mg/kg PPI; H, 8 mg/kg PPI) (* *p* < 0.05, ** *p* < 0.01, *** p < 0.001)

To determine whether PPI inhibited tumor growth in vivo, we examined it effect on Hep3B-derived tumors in BALB/c mice (Fig. 8A). Following an intraperitoneal injection of PPI (4 or 8 mg/kg) once daily for 28 d, PPI significantly prevented body weight loss induced by tumors (Fig. 8B). Furthermore, consistent with our in vitro findings, PPI treatment led to a substantial reduction in tumor volume and tumor weight in the xenograft model mice compared with those of the control group (Fig. 8C–E). Pathological examination by HE staining showed that the xenograft tumor cells were packed loosely with small nuclei compared with that observed in the control group (Fig. 8F). In the IHC assays, PPI enhanced ZBTB16 but strongly suppressed the Ki-67, indicating that PPI contributed to the major effect on tumor cell survival (Fig. 8F–H). Meanwhile, PPI induced the up-regulation of TUNEL-positive cells, confirming PPI triggering significant apoptosis in the tumor cells (Fig. 8F–I). Additionally, to evaluate the side effects of PPI, the histology of the liver and kidney were monitored and recorded. Our results showed no observable damage to the liver and kidney in PPI-treated mice (Fig. 8F). This finding was confirmed by histological analysis of the major organs, which showed no observable difference between the control and treated groups.

We next examined the effects of PPI on signaling activity in the transplanted tumors. In line with the in vitro results, ZBTB16 and PPARγ, RXRα protein levels were both increased (Fig. 8J, K), which confirmed that PPI effectively inhibits tumor growth by targeting ZBTB16 to activate the PPARγ/RXRα signaling pathway.

## Discussion

HCC is one of the most common malignant tumors in clinical practice and is associated with an extremely high incidence and mortality rate [3]. Therefore, the development of effective therapeutic agents for the treatment of HCC has always been a promising strategy. TCM has demonstrated substantial clinical benefits, playing a crucial role in the prevention and management of liver precancerous conditions, serving as an adjuvant therapy for HCC, and preventing post-surgical recurrence [32–35]. For example, clinical studies have confirmed the efficacy of Huaier Granules when used adjunctively after surgical resection and in combination with TACE, significantly reducing HCC recurrence rates [33, 34]. Similarly, numerous studies have shown that PPI has promising potential for treating various cancers [36–39]. However, the mechanism of its role for treating and slowing down the progression of HCC remains elusive and there remains an urgent need for further in-depth research.

The present study demonstrated that PPI inhibited the progression of HCC in vivo and in vitro by inhibiting proliferation, inducing apoptosis, and preventing migration and invasion. Furthermore, PPI showed no apparent toxicity to the liver and kidney of tumor-bearing mice at effective doses, preliminarily indicating its safety. Notably, the clinical use of PPI is currently problematic because of its potential adverse effects [40–43]. Although an 8 mg/kg dose in our in vivo experiments did not result in toxicity, a recent study reported that higher doses may produce adverse effects [43].

ZBTB16 was identified as the critical binding target protein of PPI against HCC in this study. It has been identified as a tumor suppressor gene of HCC, breast cancer, gastric cancer, and prostate cancer [16, 44–46], in which the mechanism of ZBTB16-mediated tumor inhibition was mainly related to cell cycle arrest, apoptosis induction, and EMT inhibition [14, 44, 47]. Our results showed that ZBTB16 knockdown promoted HCC cell proliferation in vitro and ZBTB16 overexpression blocked metastasis of HCC cell, which are consistent with the conclusions of existing studies. In addition, we also found that the abnormal down-regulation of ZBTB16 in HCC was strongly associated with poor prognosis, higher histological grade, and higher levels of APF. Thus, the ZBTB16 may serve as a promising biomarker and therapeutic target for the treatment of HCC patients and selective promotion of ZBTB16 remains an active area of drug development. PPI does represent a new lead compound for further design and discovery of ZBTB16 agonists. Attempts at modifying PPI to reduce its toxicity, controlled release, and using PPI format or in targeted/combinatorial therapies may help its clinical use.

To provide better insights into the full spectrum of the anti-HCC activity of PPI at the molecular level, a transcriptome in Hep3B cells was performed to confirm the key pathways involved in the response to the direct binding between the PPI and ZBTB16. The in-depth transcriptome analysis revealed that the inhibitory effects of ZBTB16 and PPI on HCC were involved in activating the PPAR signaling pathway. Besides, ZBTB16 was closely related to RXRα in the PPAR signaling pathway. It is reported that Peroxisome proliferator-activated receptor gamma (PPARγ) is mainly involved in the occurrence, progression and resistance development of HCC through regulating lipid metabolism [48–53], but its precise role remains controversial [54–56]. According to the experimental results in vivo and in vitro, we speculated that PPI promotes the combination of RXRα and PPARγ by targeting ZBTB16, leading to the inhibitory effect on the malignant phenotype of tumor cells. Moreover, combining ZBTB16 and PPARγ also played an important role in immunotherapy. It has been reported that activating the combination of the two would promote lipid biosynthesis, thereby improving the anti-tumor function of invariant natural killer T (iNKT) cells in the tumor microenvironment [57]. Briefly, ZBTB16 and RXRα/PPARγ pathway may be therapeutic for HCC.

It is important to note that ZBTB16 is not the only target of PPI, as PPI appears to interact with multiple target proteins. For example, our protein arrays showed that PPI potentially interacted with the shock transducer and activator of transcription 3 (STAT3) [58–60], mitogen-activated protein kinase 3 (MAPK3) [61–63], the HECT domain, and the Ankyrin Repeat Containing E3 Ubiquitin Protein Ligase 1 (HACE1) [64, 65], all of which are linked to cancer pathogenesis. Therefore, further studies are certainly warranted to determine the contribution of these potential PPI targets in HCC and other cancers.

## Conclusion

Briefly, our study indicated that ZBTB16 is a promising drug target for PPI against HCC. This supports the development of PPI as a promising therapeutic agent for regulating the ZBTB16/PPARγ/RXRα signaling axis, an action that has great potential to provide important clinical benefits.

### Supplementary Information

Supplementary Material 1

## Data Availability

The datasets used and/or analyzed during the current study are available from the corresponding author on reasonable request.

## Abbreviations

AFP: Alpha-fetoprotein
APL: Acute promyelocytic leukemia
AUC: Area under the curve
BCA: Bicinchoninic acid
BP: Biological process
CC: Cellular component
CCK8: Cell counting kit-8
DEGs: Differentially expressed genes
FBS: Fetal bovine serum
FC: Foldchange
GO: Gene ontology
GSEA: Gene set enrichment analysis
HACE1: HECT domain, and Ankyrin repeat containing E3 ubiquitin protein ligase 1
HCC: Hepatocellular carcinoma
IHC: Immunohistochemistry
KD: Dissociation constant
KEGG: Kyoto Encyclopedia of Genes and Genomes
LIHC: Liver hepatocellular carcinoma
MAPK3: Mitogen-activated protein kinase 3
MF: Molecular function
NC membranes: Nitrocellulose membranes
OS: Overall survival
P/S: Penicillin and streptomycin
PBS: Phosphate-Buffered Saline
PCA: Principal component analysis
PLZF: Promyelocytic leukemia zinc finger protein
PPI: Polyphyllin I
PPARγ: Peroxisome proliferator-activated receptor gamma
RARα: Retinoic acid receptor alpha
ROC: Receiver operating characteristic
RXRα: Retinoid X receptor alpha
SDS-PAGE: Sodium dodecyl sulfate–polyacrylamide gel electrophoresis
SPR: Surface plasmon resonance
STAT3: Shock transducer and activator of transcription 3
TCGA: The Cancer Genome Atlas Program
TCM: Traditional Chinese medicine
ZBTB16: Zinc finger and BTB domain-containing 16
