# Supplementary figures and images for: Polyphyllin I exerts anti-hepatocellular carcinoma activity by targeting ZBTB16 to activate the PPARγ/RXRα signaling pathway
Source: Chin Med. 2024 Aug 24;19:113. doi: 10.1186/s13020-024-00984-0 (PMC11344421; doi:10.1186/s13020-024-00984-0)

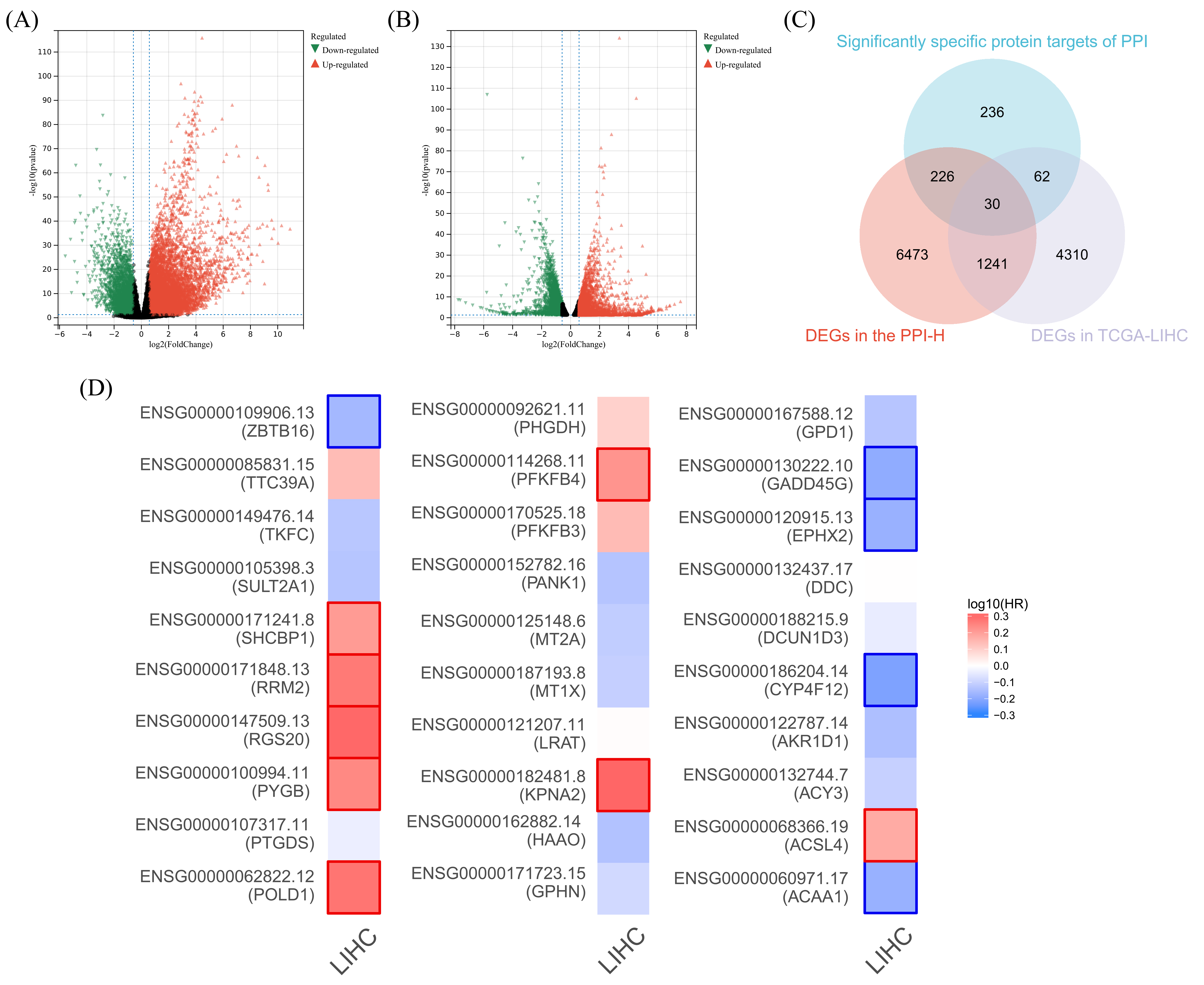

Supplement: Supplementary file 1 — Supplementary Material 1 [file 13020_2024_984_MOESM1_ESM.tif]
